# How to Make AI Ready for Rheumatology: Challenges and Perspectives

**DOI:** 10.31138/mjr.270924.rfr

**Published:** 2025-06-02

**Authors:** Eleftherios Pelechas, Panagiota G. Karagianni, Evripidis Kaltsonoudis

**Affiliations:** 1 Chatzikosta General Hospital, Department of Rheumatology, Ioannina, Greece; 2 University of Ioannina, Medical School, Department of Microbiology, Ioannina, Greece

**Keywords:** artificial intelligence, rheumatology, rheumatic diseases

In the rapidly advancing field of artificial intelligence (AI), healthcare has been identified as one of the most promising arenas for the application of AI technologies.^1^ From diagnostics to predictive analytics, AI is already making significant strides in specialties like radiology, oncology, and pathology.^2,3^ However, its integration into fields like rheumatology presents unique challenges.^4^ While AI offers tools to analyse vast datasets and assist in decision-making, its inherent limitations suggest that it is not, and cannot be, the ultimate solution to managing rheumatic diseases (RMDs). On the other hand, AI can help rheumatologists by analysing large sets of patient data to identify patterns, help to predict disease progression, and treatment plans. Furthermore, it may assist in interpreting imaging and laboratory results more accurately, leading to earlier and more precise diagnoses.

As there are already a multitude of articles about AI in rheumatology, this article will discuss, in detail, the reasons why AI cannot substitute for the nuanced, multifaceted demands of rheumatology and why it should be seen as a supportive tool rather than a replacement for the human expertise required in this specialty.

As a starting point, in **Figure 1**, a general overview of AI and its functioning is provided.

**Figure 1.**
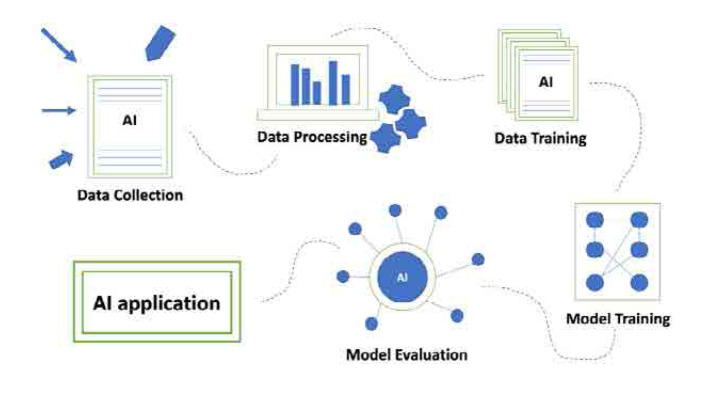
Conceptual framework for the workflow of AI development and application. The following stages are illustrated: **Data collection:** this is the initial step where raw data is gathered from various sources. This data forms the foundation for the AI system, and its quality directly impacts the system’s performance; **Data processing:** the collected data is then processed and organised into a structured format suitable for further analysis. This stage often involves cleaning the data, removing inconsistencies, and preparing it for training; **Data training:** once processed, the data is used to train AI models. This involves feeding the data into algorithms that learn patterns and relationships within the dataset; **Model training:** in this step, the AI system refines its understanding through iterative learning. This involves adjusting the model’s parameters to optimise its performance based on the training data; **Model evaluation:** after training, the model is evaluated to determine its effectiveness and accuracy. This involves testing the model on a separate dataset to ensure it generalises well to unseen data; **AI application:** finally, the trained and evaluated AI model is deployed in real-world scenarios to perform specific tasks, demonstrating its practical value. The dotted lines between stages highlight the interconnected nature of these processes, emphasising the iterative and cyclical approach often required in AI development to achieve optimal results.

## THE COMPLEXITY OF RMDs

Rheumatology deals with a diverse range of autoimmune and inflammatory conditions, including rheumatoid arthritis (RA), systemic lupus erythematosus (SLE), ankylosing spondylitis (AS), and psoriatic arthritis, among others. These diseases are characterised by their systemic nature, affecting multiple organ systems, with highly variable symptomatology. The presentation of RMDs often involves a wide range of factors: musculoskeletal pain, fatigue, cognitive dysfunction (commonly known as “brain fog”), rashes, and organ involvement such as kidney or lung disease.^5^

AI tools have been particularly effective in specialties like radiology, where image recognition tasks are well-suited for machine learning algorithms.^3^ Rheumatology, on the other hand, relies on an evolving understanding of each patient’s unique disease course. RMDs fluctuate in intensity and presentation. Symptoms may wax and wane over time, influenced by factors as varied as stress, infections, environmental triggers, and hormonal changes. These fluctuations are difficult for AI models to capture or predict, especially when they are trained on datasets that reflect static data snapshots rather than dynamic, real-world disease experiences. Here is an example for a better understanding: “*a 35-year-old woman with SLE has experienced fluctuating symptoms over the years. During periods of high stress at work, she notices increased joint pain and fatigue. Seasonal changes, particularly cold winters, tend to worsen her Raynaud’s phenomenon and cause additional discomfort. A mild respiratory infection triggers a flare with a facial rash, fever, and heightened photosensitivity. Interestingly, during pregnancy, her symptoms significantly improved due to hormonal changes, but postpartum, she suffered a severe flare*”. Such dynamic patterns make her disease experience unique and challenging to model accurately using AI, as her triggers and symptom variability depend on numerous interacting factors not easily captured by static datasets.

Furthermore, the heterogeneity of RMDs presents a significant obstacle. For example, SLE can present differently in each patient, with manifestations ranging from mild skin involvement to life-threatening nephritis or central nervous system disease. AI’s capacity to manage such diversity within a single disease category remains limited, as machine learning algorithms rely on the availability of well-structured, homogenous data—something that is lacking in rheumatology. Expanding data repositories and incorporating temporal data points could enable AI to model disease progression and individual variability more accurately.

## THE CENTRALITY OF THE DOCTOR-PATIENT RELATIONSHIP

One of the defining characteristics of rheumatology is the critical role of the doctor-patient relationship. Rheumatologists often follow patients for many years or even decades, given that many of these diseases are chronic and require long-term management. The therapeutic relationship between a rheumatologist and their patient is built on trust, empathy, and the shared experience of navigating a chronic illness.

AI, by its nature, lacks the ability to form relationships or demonstrate empathy. While AI may be able to offer diagnostic suggestions or analyse trends in a patient’s lab results, it cannot replace the deeply human aspects of patient care. Patients with chronic RMDs often struggle not only with the physical symptoms of their disease but also with the psychological and emotional toll. Depression, anxiety, and fatigue are common among patients with conditions like RA or SLE. These aspects of care require sensitivity and the ability to listen and respond to emotional as well as physical needs—an area where AI is inherently deficient.

In line with the principles of trustworthy AI, such as those promoted by the European Union AI Act, AI systems in rheumatology should respect human control and oversight, complementing rather than replacing human interaction.^6–8^ For example, decisions about initiating biologic therapies involve a thorough understanding of the patient’s lifestyle, risk tolerance, and preferences, as well as shared decision-making. These are nuances that AI, in its current state, cannot replicate, underscoring the need for AI as an assistive tool rather than a substitute for physician judgment and empathy.

## RHEUMATOLOGY RELIES ON CLINICAL JUDGMENT, NOT JUST DATA

AI excels in environments where data is plentiful, clean, and well-structured. However, rheumatology often requires clinicians to make decisions based on incomplete, messy, or ambiguous data. And we are not referring solely to clinical research, where the data is more structured, and patient characteristics are often limited by strict inclusion criteria. Instead, we are focusing on the application of this data to each individual patient we encounter, ensuring they receive the appropriate and personalised treatment they require. Furthermore, many RMDs do not have a single definitive test. The diagnosis of SLE exemplifies the complexity of clinical decision-making in rheumatology. It often requires synthesising diverse inputs-clinical symptoms, serological tests, and sometimes biopsies-all of which may present inconclusive or even conflicting results. In such cases, clinical judgment and experience are essential for interpreting these factors and arriving at a diagnosis. This variability in subjective decision-making among clinicians underscores both the strengths and limitation of machine learning (ML) models in this context. A notable aspect of clinical decision-making in SLE is its inherent subjectivity. Two clinicians may weigh the importance of clinical signs, lab results, and contextual factors differently, leading to variations in diagnoses or treatment approaches. In contrast, ML models could offer a more standardised approach, providing consistent decisions when applied to similar data inputs. This stability could reduce variability in certain aspects of care, particularly when clear patterns or thresholds are present in the data. However, the reliance of ML models on structured and clean data is a critical limitation. While these models may excel in identifying patterns or making predictions based on large datasets, they often fail to account for the nuanced, patient-specific factors that clinicians intuitively incorporate. For instance, a serological marker like ANA positivity, while a feature in diagnosing SLE, might be less relevant in isolation for a specific patient if clinical symptoms do not align, a context an experienced clinician would recognise, but an ML model might not.

Another challenge is the applicability of clinical guidelines, which are developed based on aggregated evidence and are often designed for “typical” cases. Guidelines provide a foundation for diagnosis and management, but real-world patients frequently present with atypical or overlapping features that require deviation from standard recommendations. ML models, trained on large datasets often derived from guideline-based research, might struggle to generalise to these outlier cases. For example, an ML model trained to predict SLE based on established diagnostic criteria might fail to recognise a patient with atypical symptoms or an uncommon disease trajectory.

Moreover, the patient experience of disease often involves subjective symptoms, such as fatigue or pain, that are difficult to quantify or capture through objective data points alone. For example, in diseases like fibromyalgia or early stages of spondyloarthritis, imaging and blood tests may be normal, yet patients experience significant discomfort and functional impairment. AI systems that rely solely on measurable data will likely miss these nuances, leading to potential under-treatment or mismanagement.

Where ML models could shine in complementing clinical judgment by identifying patterns that may not be immediately apparent to clinicians, such as subtle correlations in serological markers or genetic data. Additionally, they could assist in stratifying patients based on disease severity, predicting flares, or tailoring treatment plans. However, these applications must be seen as adjuncts to, rather than replacements for, clinical expertise.

## BIAS IN AI MODELS: A MAJOR CONCERN FOR RHEUMATOLOGY

AI models are only as good as the data on which they are trained, and one of the major criticisms of AI in healthcare is the risk of bias.^9–11^ Rheumatology in particular faces a significant challenge in this area. RMDs are known to affect different ethnic and racial groups in distinct ways. For example, African American, Hispanic, and Asian patients have higher rates of severe disease in conditions like SLE.^12^ However, many AI models are trained predominantly on data derived from white, European-ancestry populations. As a result, these models may not generalise well to underrepresented groups, exacerbating healthcare disparities.^13^

Furthermore, RMDs are more prevalent in women,^14^ who are often underrepresented in clinical trials and research datasets.^15^ If AI models are trained on biased data, they may fail to recognise atypical presentations or respond appropriately to patient-specific factors like sex or ethnicity.^16^ The reliance on biased AI models could lead to worse outcomes for marginalised populations, which is a significant ethical concern in a field where individualised care is paramount.^17^

The last but of extreme importance is the issue of “false results in research”. The issue of “false results in research” becomes critically important when considering the integration of AI into medical decision-making. Professor Ioannidis’ essay, “Why Most Published Research Findings are False”,^18^ highlights the prevalence of biases, small sample sizes, and methodological flaws that contribute to unreliable findings in scientific literature. When AI systems rely on such flawed data, significant problems can arise. AI models are only as reliable as the data they are trained on. At this stage, it’s important to refer back to **Figure 1**: the initial step in the process is data collection. Regardless of how advanced the algorithms or system training become, the outcome will inevitably be influenced by inherent biases. If these systems are built using biased or false findings, the inaccuracies may be amplified, leading to misleading conclusions or suboptimal recommendations. For instance, an AI trained on studies with exaggerated effect sizes or poorly controlled trials may generate treatment suggestions that fail in real-world clinical practice. Additionally, AI models might overlook context-specific nuances, such as population differences or unmeasured confounders, further compounding the issue. The lack of transparency in AI algorithms poses another challenge.^19^ When a model’s recommendations are influenced by flawed research, it can be difficult for clinicians to identify and correct the errors. This could erode trust in AI tools and potentially harm patients. To address these concerns, rigorous validation of AI models using high-quality, real-world data is essential. Robust oversight and continuous monitoring will ensure AI systems align with evidence-based, reliable practices.

## CHALLENGES OF LONG-TERM DISEASE MANAGEMENT

Rheumatology is not solely about diagnosing diseases but also about managing them effectively over the long term with the suitable drug strategies.^20^ This requires rheumatologists to frequently adjust treatment regimens in response to evolving disease activity, medication side effects, patient preferences, and emerging clinical evidence. Decisions to escalate or taper treatment are often influenced by subtle cues (both objective clinical findings and subjective patient-reported symptoms) that may not always align. For instance, a rheumatologist might decide to modify treatment based on a patient’s report of early morning stiffness or fatigue, even if laboratory values remain within normal limits. Conversely, AI systems, which typically prioritise objective data such as laboratory results, might undervalue or overlook these subjective cues, potentially leading to decisions that do not fully address the patient’s condition or quality of life.

However, there is insufficient evidence to definitively determine whether a human-centred, subjective approach or a data-driven, objective AI approach is inherently superior or inferior. While subjective symptoms such as fatigue and pain provide essential insights into a patient’s lived experience, they can be influenced by non-disease factors such as psychological state, stress, or comorbidities. This variability can make them less reliable as standalone indicators of disease activity. Conversely, while laboratory markers and objective data are consistent and quantifiable, they may not always correlate directly with the patient’s symptom burden or overall well-being. For example, a patient may experience significant discomfort despite normal inflammatory markers, or vice versa.^21^

The art of rheumatology lies in integrating both approaches. Clinicians bring the ability to interpret subjective symptoms within the context of the patient’s broader clinical picture, while AI offers consistency and scalability, processing vast amounts of data to identify patterns and trends. A “hybrid” model, where AI supports decision-making by providing evidence-based recommendations but allows clinicians to weigh subjective and contextual factors, could be the most effective path forward.

Future research should focus on assessing how AI can be designed to incorporate patient-reported outcomes alongside objective clinical data. Long-term studies comparing outcomes from AI-driven versus clinician-driven decisions, or hybrid approaches, could provide valuable insights. Until then, rheumatology remains reliant on the human ability to balance science with empathy, an art that AI, at its current level of development, cannot yet replicate.^22^ This underscores the importance of collaboration between technology and clinical expertise in achieving optimal patient care.

## ETHICAL AND ACCOUNTABILITY ISSUES IN AI-DRIVEN DECISION MAKING

The integration of AI in clinical decision-making raises ethical concerns, particularly regarding responsibility and accountability.^23^ In rheumatology, the consequences of misdiagnosis or inappropriate treatment can be severe, leading to irreversible joint damage, organ failure, or a reduced quality of life. If AI is involved in making clinical decisions, who is accountable when something goes wrong? Is it the physician who relied on the AI tool, or the developers of the AI system?

AI models are also often “black boxes”, meaning their decision-making processes are opaque.^24^ Physicians may not understand exactly how an AI tool arrived at a particular recommendation, yet they are expected to trust the result. In a field like rheumatology, where individualised care and clinical intuition play significant roles, this lack of transparency is particularly problematic.

## AI AS A COMPLEMENTARY TOOL, NOT A REPLACEMENT

Despite its limitations, AI has the potential to play a supportive role in rheumatology. For instance, AI can be useful in analysing large datasets to identify trends, predict disease flares, or assist in drug discovery.^25^ It can also help streamline certain administrative tasks, such as documenting patient encounters or sifting through electronic health records to identify pertinent information.

AI can complement the expertise of rheumatologists by serving as a diagnostic aid or by providing risk assessments based on large datasets. However, its role should remain complementary, rather than central.^26^ The complexity of RMDs, the importance of the doctor-patient relationship, and the need for nuanced clinical judgment ensure that rheumatology will continue to rely primarily on human expertise.

## CONCLUSION: AI IS NOT THE PANACEA FOR RHEUMATOLOGY

AI undoubtedly holds great promise in healthcare, but rheumatology poses unique challenges that AI is not equipped to overcome on its own. At this point, it is important to highlight the distinction between the applicability of AI in chronic diseases broadly and its specific challenges in rheumatology. While AI holds significant promise in areas such as diagnosis, prognosis, and predicting treatment responses, the unique complexity of rheumatic and musculoskeletal diseases makes its role inherently different in rheumatology.

In chronic diseases, AI excels at identifying patterns, stratifying patients by risk, and optimising treatment pathways based on large datasets. For example, in oncology, AI has shown success in predicting treatment outcomes by analysing genetic profiles. Similarly, in cardiology, it aids in early detection of conditions through imaging and wearable device data. These successes highlight AI’s potential to revolutionise chronic disease care by leveraging data-rich environments and structured datasets.

However, the challenges in rheumatology go beyond these capabilities. RMDs often involve subjective symptoms like pain, fatigue, and stiffness, which are difficult for AI to quantify or interpret accurately. Furthermore, the centrality of the patient-physician relationship and the need for nuanced clinical judgment, particularly in cases with conflicting data or atypical presentations, are areas where AI falls short. Additionally, the heterogeneity of RMDs and the absence of definitive biomarkers for many conditions limit the utility of data-drive AI models in this field.

Ethical considerations, such as the risk of bias in training data and the potential for AI to depersonalise care, also weigh heavily in rheumatology. While AI can enhance aspects like data analysis and administrative efficiency, it cannot replicate the empathy, intuition, and adaptability of a skilled rheumatologist.

Thus, the future of rheumatology should focus on integrating AI as an assistive tool that complements clinical expertise. By combining AI’s analytical power with the human elements on medicine, rheumatology can harness the best of both worlds to improve patient outcomes while preserving the art of personalised care. **Table 1** depicts challenges in Rheumatology and the possible AI approaches.

**Table 1.** Sample characteristics.

**Challenge in Rheumatology**	**Description**	**AI approaches to overcome challenge**
**Early diagnosis of RMDs**	Many rheumatic diseases have overlapping symptoms, making early diagnosis difficult	Machine learning algorithms for pattern recognition in imaging, EHR analysis, and predictive modelling
**Patient stratification**	Identifying subgroups of patients who may respond differently to treatments is essential for personalised medicine	Clustering and classification algorithms based on clinical, genetic, and lifestyle data
**Predicting disease progression**	Chronic rheumatic diseases can progress unpredictably, complicating treatment planning	Predictive modelling using time-series analysis and deep learning on patient data over time
**Treatment response prediction**	Variable patient responses to treatments make it challenging to choose the right therapy initially	Machine learning models to predict treatment efficacy based on genetic, phenotypic, and lifestyle data
**Data integration from diverse sources**	Data sources in rheumatology include clinical notes, imaging, genetics, and wearable devices, which are difficult to integrate	NLP for EHR and clinical notes, multi-modal learning for cross-source data integration
**Patient adherence and monitoring**	Adherence to treatment is crucial but hard to monitor, especially in chronic diseases	AI-powered mobile health apps, wearable technology, and predictive analytics to track and predict adherence
**Optimising drug development**	Developing drugs for rheumatic diseases is costly and time-consuming	AI-driven drug discovery, molecular modelling, and virtual screening of potential compounds

## CONFLICT OF INTEREST

The authors declare no conflict of interest.
